# Drug Utilization Patterns and WHO Prescribing Indicators Among Hypertensive Outpatients at Karnali Academy of Health Sciences, Nepal: A Cross‐Sectional Study

**DOI:** 10.1002/prp2.70341

**Published:** 2026-11-01

**Authors:** Bijay Aryal, Birendra Kumar Yadav, Biswash Sapkota, Surakshya Adhikari

**Affiliations:** ^1^ Department of Clinical Pharmacology School of Health Sciences, Martyr Dasharath Chand University of Health Science Geta, Kailali Nepal; ^2^ School of Pharmacy Karnali Academy of Health Sciences Jumla Nepal; ^3^ Department of Pharmacy Madan Bhandari Academy of Health Sciences Hetauda Nepal; ^4^ Department of Biochemistry Pokhara Academy of Health Sciences Pokhara Nepal

**Keywords:** drug utilization, hypertension, Nepal, prescribing, rational, WHO prescribing indicators

## Abstract

Hypertension is one of the most prevalent non‐communicable diseases (NCDs) worldwide. This descriptive cross‐sectional study evaluated drug utilization patterns and WHO core prescribing indicators among hypertensive patients attending the Outpatient Department of Karnali Academy of Health Sciences (KAHS), Jumla. A total of 197 hypertensive outpatients were systematically enrolled, and data were collected using a structured WHO‐aligned questionnaire and analyzed using descriptive statistics in SPSS version 26. The mean age of participants was 51.6 ± 13.2 years, with a slight male predominance (51.3%). Calcium channel blockers (32.8%) and angiotensin receptor blockers (24.6%) were the most frequently prescribed antihypertensive classes, while dual‐drug therapy was the predominant treatment regimen (43.7%). Assessment of WHO prescribing indicators showed an average of 4.05 drugs per prescription (WHO target < 2.0), generic prescribing of 34.4% (target 100%), adherence to the Essential Medicines List of 79.9% (target 100%), antibiotic prescribing of 14.2%, and injection prescribing of 7.6%. Although antihypertensive prescribing practices at KAHS reflected generally appropriate drug selection for hypertension management, substantial deviations from WHO rational medicine use standards were observed, particularly with regard to low generic prescribing and excessive polypharmacy. These findings highlight the need for urgent policy‐level interventions, including mandatory generic prescribing practices and regular prescription audits, to improve affordability, promote rational drug use, and enhance patient safety in this resource‐limited setting.

## 1 Introduction

Hypertension (HTN) is defined as a systolic blood pressure (BP) ≥ 140 mmHg and/or diastolic blood pressure ≥ 90 mmHg [1]. It is one of the most prevalent non‐communicable diseases (NCDs) worldwide and represents a major public health challenge due to its strong association with cardiovascular, cerebrovascular, renal, and ocular complications. The pathological effects of hypertension primarily involve damage to the blood vessels of vital organs, including the heart, brain, kidneys, and eyes. Hypertension is also recognized as one of the most important modifiable risk factors for stroke, heart failure, myocardial infarction, and chronic kidney disease [2].

According to the World Health Organization (WHO), hypertension contributes to approximately 9.4 million deaths annually and accounts for nearly 7% of global Disability‐Adjusted Life Years (DALYs) [3]. Globally, hypertension ranks as the fourth leading risk factor for premature mortality in developed countries and the seventh in developing countries [4]. The burden is disproportionately higher in low‐ and middle‐income countries (LMICs), where access to early diagnosis, long‐term treatment, and continuous monitoring remains limited and frequently unaffordable [5]. Between 2011 and 2025, the cumulative loss of socioeconomic productivity attributable to NCDs, including hypertension, is estimated to reach USD 7.28 trillion [6]. In LMICs, pharmaceutical expenditures constitute approximately 30%–40% of total healthcare spending, and cost‐related non‐adherence to antihypertensive therapy is highly prevalent [7, 8].

The principal pharmacological classes used in the management of hypertension include thiazide diuretics, beta‐blockers (BBs), angiotensin‐converting enzyme inhibitors (ACEIs), angiotensin receptor blockers (ARBs), and calcium channel blockers (CCBs) [9, 10, 11]. Evidence‐based treatment guidelines such as the Joint National Committee (JNC 7 and JNC 8) and WHO recommendations advocate thiazide diuretics as first‐line therapy for uncomplicated hypertension because of their proven efficacy, safety, and cost‐effectiveness [12, 13, 14]. However, recent evidence indicates that prescribing practices have increasingly shifted toward newer and more expensive agents such as ACEIs and CCBs, often at the expense of older generic alternatives [3, 15, 16]. Such prescribing trends may not only contradict established treatment guidelines but also increase treatment costs and contribute substantially to the economic burden of hypertension management [17, 18].

Drug Utilization Research (DUR), defined by the WHO as the study of the marketing, distribution, prescription, and use of drugs in society with particular emphasis on their medical, social, and economic consequences, is an essential tool for evaluating prescribing quality and promoting rational medicine use [17]. DUR plays a critical role in identifying irrational prescribing patterns such as polypharmacy, inappropriate drug selection, unnecessary use of brand‐name medicines, and cost‐inefficient therapy [19, 20]. To facilitate standardized evaluation of prescribing practices, the WHO developed core drug use indicators, including prescribing, patient‐care, and facility indicators, which are widely used to assess rational drug use in healthcare systems globally [21, 22, 23].

The prevalence of hypertension has increased rapidly across South Asia, including India and Nepal, driven by urbanization, dietary transition, sedentary lifestyle, obesity, and high salt consumption [24]. In India, the prevalence of hypertension is estimated at approximately 29%, while cardiovascular diseases account for nearly 1.5 million deaths annually [25, 26]. Hypertension contributes to approximately 57% of stroke‐related deaths and 24% of coronary heart disease deaths in the country [27]. Despite the availability of national and international treatment guidelines, several studies have documented irrational prescribing patterns among hypertensive patients, including excessive use of expensive ACEIs and CCBs, underutilization of diuretics, inappropriate dosing, and poor adherence to evidence‐based recommendations [13, 16]. Factors such as pharmaceutical marketing influence, inadequate awareness of drug costs, and limited implementation of generic prescribing policies have been implicated in these practices [28].

Nepal is experiencing a growing burden of hypertension and related cardiovascular diseases, with national estimates indicating that approximately 24% of men and 17% of women aged 15 years and above are hypertensive, while some regional studies report even higher prevalence rates [29, 30]. Cardiovascular diseases are among the leading causes of mortality in Nepal, and hypertension remains the most significant modifiable risk factor [27]. Multiple determinants—including tobacco use, unhealthy diet, physical inactivity, alcohol consumption, stress, low health literacy, and inadequate screening—contribute to low diagnosis and treatment rates, particularly in remote and underserved regions such as Jumla, where geographic barriers, limited healthcare infrastructure, and economic constraints further compromise access to essential medicines and quality care [11, 29]. Although Nepal has adopted national treatment guidelines aligned with WHO and JNC recommendations [13], irrational prescribing practices (e.g., excessive use of branded medications, inappropriate dosing, and polypharmacy) remain common [30]. Medicines account for approximately 40% of the national health budget, underscoring the importance of rational use from both public health and economic perspectives [7]. The WHO introduced the Essential Medicines concept in 1977 to ensure equitable access to safe, effective, and affordable medicines for priority healthcare needs [31, 32]; however, ensuring consistent availability and affordability of essential antihypertensive medicines remains a significant challenge in rural and geographically isolated regions of Nepal.

In such settings, prescription audits and drug utilization studies are essential for evaluating prescribing quality, adherence to treatment guidelines, and affordability of therapy [31, 32]. Karnali Academy of Health Sciences (KAHS), located in Jumla district, serves as a major tertiary healthcare institution for a large underserved population in Karnali Province. Despite the growing burden of hypertension in the region, evidence regarding antihypertensive prescribing practices and rational drug use at KAHS remains limited. Therefore, evaluating drug utilization patterns and WHO prescribing indicators among hypertensive patients attending the outpatient department of KAHS is essential to generate context‐specific evidence that can guide interventions aimed at improving prescribing quality, promoting rational drug use, reducing unnecessary healthcare expenditure, and enhancing blood pressure control and patient outcomes in remote healthcare settings.

## 2 Methods

### 2.1 Study Design, Study Period, and Site

This study employed a descriptive cross‐sectional design within a hospital outpatient setting of Karnali Academy of Health Sciences (KAHS), Jumla, Nepal. It lies in the 29°27′ N latitude and 82°17′ E longitude. The KAHS was established to enable access to quality healthcare services and education for the people of backward areas at an affordable cost [33]. KAHS serves as the sole tertiary‐care academic hospital in Karnali Province and functions as the primary referral center for the region. The study was conducted for the period from June 2025 to December 2025.

### 2.2 Study Population

The study population comprised adult hypertensive patients (aged ≥ 18 years) attending the KAHS Outpatient Department (OPD) during the data collection period who provided informed consent and were prescribed at least one antihypertensive medication. This included both patients receiving ongoing antihypertensive treatment and those initiating therapy for the first time, enabling the assessment of routine prescribing practices in a real‐world, resource‐limited healthcare setting. Patients receiving inpatient care or treatment in the emergency department were excluded. Additionally, individuals with significant cognitive impairment, communication difficulties, or psychiatric conditions that could interfere with their ability to participate in the study at the time of data collection were not included.

### 2.3 Sample Size

The sample size was calculated using Cochran's formula for estimating sample size. A confidence level of 95% was used, corresponding to a *Z*‐score of 1.96. The estimated proportion (*p*) of 0.142 was observed from Khanal et al. [34], *q* (1 − *p*) was 0.858. The margin of error (*e*) was set at 5% (0.05). Using Cochran's formula, the sample size was calculated as 187.4. To account for a possible non‐response rate of 5%, the sample size was adjusted to 197 hypertensive patients.

### 2.4 Data Collection Tool

The questionnaire was developed in English after reviewing previously published studies of a similar nature, with necessary modifications made to align with the objectives and context of the present study. Following its development, the questionnaire underwent a review and validation process to ensure its suitability, clarity, and accuracy. A pre‐test was conducted among 18 hypertensive patients attending the outpatient department (approximately 10% of the calculated sample size of 197) to assess the readability, comprehensibility, and overall acceptability of the questionnaire. Face validation was performed by a professor from the Department of Pharmacy, while subject experts from the medical department and a statistician were consulted to evaluate the content validity and structure of the instrument. The internal consistency and reliability of the questionnaire were assessed using Cronbach's alpha, which yielded a value of 0.70, indicating acceptable reliability and consistency of the questionnaire items. Data obtained during the pre‐testing phase were excluded from the final analysis; however, feedback from the pre‐test was used to refine the questionnaire through the rephrasing of sentences and improvements in grammar, wording, and overall clarity.

The final version of the data collection questionnaire comprised five sections, each designed to capture specific aspects of antihypertensive drug utilization patterns. Section 1 collected sociodemographic information and consisted of six items. Section 2 assessed the clinical profile of the patients through 10 items, while Section 3 focused on prescription‐related information and included seven items. Section 4 examined antihypertensive drug utilization and was divided into two subsections: the first subsection assessed the therapy pattern using a single item, and the second subsection consisted of six items related to antihypertensive medication use, Section 5 evaluated WHO prescribing indicators and contained five items.

### 2.5 Data Collection Technique

A prospective cross‐sectional data collection approach was employed in this study. After obtaining informed consent, the researcher reviewed each participant's outpatient prescription issued during the same OPD visit and recorded the relevant information directly onto a structured data collection proforma. This method facilitated the real‐time capture of prescribing practices, minimized the risk of recall bias, and enabled efficient data collection without disrupting routine clinical services or patient care.

### 2.6 Statistical Analysis

Data collected was entered into Microsoft Excel 2019 using a double‐entry verification method to ensure accuracy. The dataset was then cleaned by checking for inconsistencies, missing values, and outliers, followed by coding and organization to prepare a complete database for analysis. Statistical analysis was performed using IBM SPSS Statistics version 26.0. Descriptive statistics (frequency, percentage, mean, and standard deviation) were used to summarize sociodemographic characteristics, drug utilization patterns, and WHO prescribing indicators. Inferential analysis was conducted using the Chi‐square test to assess associations between categorical variables such as JNC‐7 stage, age group, and antihypertensive therapy patterns, with a *p*‐value of < 0.05 considered statistically significant.

## 3 Results

### 3.1 Sociodemographic Characteristics

A total of 197 participants were included in the study. Overall, the sample shows a fairly balanced gender distribution, with slightly more males (51.3%) than females (48.7%). Most participants were in the middle to older age groups, with the highest proportion in the 50–59 years' category (23.9%), indicating that hypertension was more common in middle‐aged and elderly individuals.

Regarding education, a considerable proportion had secondary‐level education (39.1%), while a notable segment was illiterate (22.3%), suggesting moderate educational attainment in the study population. In terms of occupation, housewives represented the largest group (37.6%), followed by farmers (28.4%), reflecting a predominantly non‐service‐based population. Most participants were married (84.3%), and a substantial proportion (64.0%) reported a family history of hypertension, indicating a strong familial predisposition (Table 1).

**TABLE 1:** Socio‐demographic characteristics of patients (*N* = 197).

Variables	Frequency (%)
Sex	
Male	101 (51.3)
Female	96 (48.7)
Age group	
18–29	14 (7.1)
30–39	32 (16.2)
40–49	42 (21.3)
50–59	47 (23.9)
60–69	38 (19.3)
≥ 70	24 (12.2)
Educational level	
Illiterate	44 (22.3)
Primary (1–5)	40 (20.3)
Secondary (6–10)	77 (39.1)
Higher (+2 & above)	36 (18.3)
Occupational distribution	
Farmer	56 (28.4)
Service	15 (7.6)
Business	26 (13.2)
Housewife	74 (37.6)
Student	15 (7.6)
Unemployed	11 (5.6)
Marital status	
Married	166 (84.3)
Unmarried	19 (9.6)
Widowed	5 (2.5)
Separated	7 (3.6)
Family history of hypertension	
Present	126 (64.0)
Absent	70 (35.5)
Unknown	1 (0.5)
Smoking	
Yes	35 (17.8)
No	162 (82.2)
Alcohol consumption	
Yes	46 (23.4)
No	151 (76.6)
Physical inactivity	
Yes	150 (76.1)
No	47 (23.9)
High salt intake	
Yes	186 (94.4)
No	11 (5.6)

Lifestyle characteristics revealed a low prevalence of smoking (17.8%) and alcohol consumption (23.4%). However, a large majority of participants were physically inactive (76.1%) and reported high salt intake (94.4%), highlighting significant modifiable risk factors within the study population.

### 3.2 Clinical Characteristics

The mean duration of hypertension was 7.97 ± 5.70 years (range: 0.5–25 years), and more than half of the participants (106; 53.8%) had been diagnosed for over 5 years. The mean systolic blood pressure was 155.91 ± 12.98 mmHg (range: 118–182 mmHg), while the mean diastolic blood pressure was 95.27 ± 6.46 mmHg (range: 76–108 mmHg). Regarding blood pressure classification as per JNC‐7, Stage 1 hypertension was most common (45.2%), closely followed by Stage 2 hypertension (44.7%). Pre‐hypertension was observed in 19 (9.6%) participants, and only 1 (0.5%) participant had normal blood pressure (Table 2).

**TABLE 2:** JNC‐7 classification of hypertension (*N* = 197).

JNC‐7 category	BP range (mmHg)	Frequency (%)	Percentage (%)
Normal	< 120/< 80	1	0.5
Pre‐hypertension	120–139/80–89	19	9.6
Stage 1 hypertension	140–159/90–99	89	45.2
Stage 2 hypertension	≥ 160/≥ 100	88	44.7

The largest proportion of patients were overweight, comprising 81 (41.1%) of the study population. This was followed by those classified as obese Class I, accounting for 50 (25.4%) participants. Normal BMI was observed in 45 (22.8%) patients. Smaller proportions of participants were categorized as obese Class II (12; 6.1%), obese Class III (4; 2.0%), and underweight (5; 2.5%) (Table 3).

**TABLE 3:** BMI classification of patients (*N* = 197).

BMI category	BMI range (kg/m^2^)	Frequency (*n*)
Underweight	< 18.5	5 (2.5)
Normal	18.5–24.9	45 (22.8)
Overweight	25.0–29.9	81 (41.1)
Obese Class I	30.0–34.9	50 (25.4)
Obese Class II	35.0–39.9	12 (6.1)
Obese Class III	≥ 40.0	4 (2.0)

Diabetes mellitus was the most common comorbidity, present in 22.8% of participants, while gout was the least common, observed in only 1.0% of patients, as shown in Table 4.

**TABLE 4:** Frequency of comorbid conditions among hypertensive patients.

Comorbidity	Frequency (*n*)	Percentage (%)
Diabetes mellitus	45	22.8
Dyslipidemia	39	19.8
Chronic kidney disease	23	11.7
Ischemic heart disease	18	9.1
Congestive heart failure	17	8.6
Angina	17	8.6
Peptic ulcer disease	17	8.6
Urinary tract infection	16	8.1
Anemia	13	6.6
Stroke	10	5.1
Asthma/COPD	8	4.1
Myocardial infarction	6	3.0
Other comorbidities	6	3.0
Seizure disorder	5	2.5
Gout	2	1.0

### 3.3 Drug Utilization Patterns

A total of 799 prescribed drugs among hypertensive patients were categorized according to their therapeutic class. Antihypertensive drugs were the most frequently prescribed, accounting for 44.7% of all medications, followed by other cardiovascular drugs (8.0%) and anti‐ulcer drugs (8.0%). Lipid‐lowering drugs (7.8%), antidiabetic drugs (5.9%), and vitamins and supplements (5.3%) were also commonly prescribed. Respiratory and central nervous system drugs each accounted for 1.4%, while analgesics, antiplatelet drugs, and non‐antihypertensive diuretics were each prescribed in only 0.9% of cases as shown in Table 5.

**TABLE 5:** Distribution of all prescribed drugs by therapeutic category among hypertensive patients (*N* = 799).

Therapeutic category	Number of prescriptions	Percentage (%)
Antihypertensive drugs	357	44.7
Other cardiovascular drugs	64	8.0
Anti‐ulcer drugs	64	8.0
Lipid‐lowering drugs	62	7.8
Antidiabetic drugs	47	5.9
Vitamins and supplements	42	5.3
Antibiotics	28	3.5
Non‐steroidal anti‐inflammatory drugs (NSAIDs)	23	2.9
Respiratory drugs	11	1.4
Central nervous system drugs	11	1.4
Analgesics	7	0.9
Antiplatelet drugs	7	0.9
Diuretics (non‐antihypertensive)	7	0.9
Other drugs^a^	69	8.4
	799	100.0

^a^ Other drugs include miscellaneous therapeutic categories with individual frequencies < 5, including gastrointestinal drugs, drugs for gout, hematinics, and various supplements.

Among 357 antihypertensive drugs prescribed, CCBs were the most frequent (32.8%), followed by ARBs (24.6%), diuretics (19.3%), beta‐blockers (17.6%), and ACE inhibitors (5.6%) as the least prescribed as shown in Figure 1.

**FIGURE 1:**
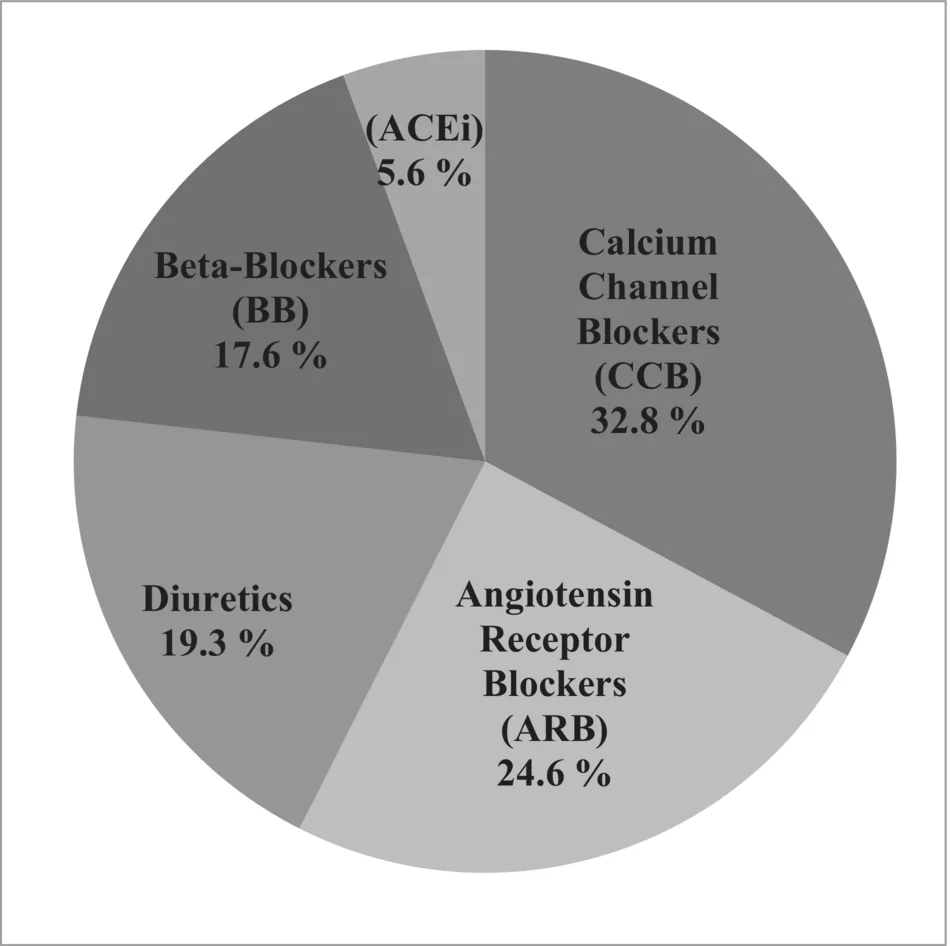
Distribution of antihypertensive drugs by pharmacological class (*n* = 357).

Among CCBs, amlodipine was most prescribed (92.3%), followed by nifedipine (4.3%) and clinidipine (3.4%). In ARBs, telmisartan was most common (69.3%), followed by losartan (29.5%) and olmesartan (1.1%). Among diuretics, furosemide (40.6%) and hydrochlorothiazide (39.1%) were most frequent, followed by spironolactone (18.8%) and indapamide (1.4%). Atenolol was the predominant beta‐blocker (76.2%), followed by propranolol and bisoprolol (6.3%), metoprolol and carvedilol (4.8%), and nebivolol (1.6%). Within ACE inhibitors, ramipril was most common (95.0%), while enalapril accounted for 5.0% prescription (Table 6).

**TABLE 6:** Distribution of antihypertensive drugs by class and generic name (*n* = 357).

Pharmacological class	Drug name	Frequency (*n*)	Percentage within class (%)
Calcium channel blockers (CCB)		117	100.0
	Amlodipine	108	92.3
	Nifedipine	5	4.3
	Clinidipine	4	3.4
Angiotensin receptor blockers (ARB)		88	100.0
	Telmisartan	61	69.3
	Losartan	26	29.5
	Olmesartan	1	1.1
Diuretics		69	100.0
	Furosemide	28	40.6
	Hydrochlorothiazide	27	39.1
	Spironolactone	13	18.8
	Indapamide	1	1.4
Beta‐blockers (BB)		63	100.0
	Atenolol	48	76.2
	Metoprolol	3	4.8
	Propranolol	4	6.3
	Bisoprolol	4	6.3
	Carvedilol	3	4.8
	Nebivolol	1	1.6
ACE inhibitors (ACEi)		20	100.0
	Ramipril	19	95.0
	Enalapril	1	5.0

Table 7 presents a detailed list of antihypertensive drugs prescribed in the study along with their Defined Daily Dose (DDD) analysis based on the WHO Anatomical Therapeutic Chemical (ATC) classification. Among the antihypertensive drugs, amlodipine was the most frequently prescribed agent, accounting for 108 (30.3%) prescriptions and a total of 3390 DDDs. This was followed by telmisartan with 61 (17.1%) prescriptions corresponding to 1890 DDDs, and atenolol with 48 (13.4%) prescriptions and 980 DDDs. Losartan and furosemide accounted for 810 and 870 DDDs, respectively. Hydrochlorothiazide contributed 405 DDDs, while spironolactone accounted for 260 DDDs. Ramipril showed a total of 1140 DDDs despite a relatively lower number of prescriptions. Other antihypertensive agents, including nifedipine, clinidipine, propranolol, bisoprolol, metoprolol, carvedilol, nebivolol, olmesartan, enalapril, and indapamide, contributed smaller proportions to the overall drug utilization. The cumulative total DDDs for all antihypertensive drugs prescribed during the study period was 10 163.

**TABLE 7:** Detailed list of antihypertensive drugs with DDD analysis.

Drug name	(*n*)	(%)	ATC code	S mg	T tabs	T mg	D mg	DDDs
Amlodipine	108	30.3	C08CA01	5	3390	16 950	5	3390.0
Telmisartan	61	17.1	C09CA07	40	1890	75 600	40	1890.0
Atenolol	48	13.4	C07AB03	50	1470	73 500	75	980.0
Losartan	26	7.3	C09CA01	50	810	40 500	50	810.0
Furosemide	28	7.8	C03CA01	40	870	34 800	40	870.0
Hydrochlorothiazide	27	7.6	C03AA03	12.5	810	10 125	25	405.0
Spironolactone	13	3.6	C03DA01	25	390	9750	37.5	260.0
Ramipril	19	5.3	C09AA05	5	570	2850	2.5	1140.0
Nifedipine	5	1.4	C08CA05	10	165	1650	30	55.0
Clinidipine	4	1.1	C08CA14	10	120	1200	10	120.0
Propranolol	4	1.1	C07AA05	40	120	4800	160	30.0
Bisoprolol	4	1.1	C07AB07	5	120	600	10	60.0
Metoprolol	3	0.8	C07AB02	50	90	4500	150	30.0
Carvedilol	3	0.8	C07AG02	12.5	90	1125	37.5	30.0
Nebivolol	1	0.3	C07AB12	5	30	150	5	30.0
Olmesartan	1	0.3	C09CA08	20	30	600	20	30.0
Enalapril	1	0.3	C09AA02	5	30	150	10	15.0
Indapamide	1	0.3	C03BA11	1.5	30	45	2.5	18.0
Total	357	100.0			11 025	278 895		10163.0

*Note:* DDDs calculated as: total amount (mg) ÷ WHO DDD (mg).

Abbreviations: ATC = anatomical therapeutic chemical classification, DDD = defined daily dose, D mg = WHO defined daily dose in milligrams, *n* = frequency, S mg = strength in milligrams, T mg = total amount in milligrams, T tabs = items issued in tablets.

### 3.4 Antihypertensive Therapy Patterns

Dual therapy was the most commonly prescribed regimen, accounting for 86 (43.7%) participants, followed by monotherapy in 77 (39.1%) participants. Triple or more drug therapy was prescribed in 34 (17.2%) participants. The mean number of antihypertensive drugs prescribed per patient was 1.82 ± 0.87, with the number of drugs ranging from 1 to 4 as shown in Table 8.

**TABLE 8:** Antihypertensive therapy patterns among study participants (*N* = 197).

Therapy type	Number of patients	Percentage (%)	95% confidence interval
Monotherapy	77	39.1	(32.3–46.3)
Dual therapy	86	43.7	(36.7–50.9)
Triple therapy	31	15.7	(11.1–21.6)
Quadruple therapy	3	1.5	(0.3–4.4)

Table 9 presents the specific combinations of antihypertensive drugs used in monotherapy, dual therapy, triple therapy, and quadruple therapy. Among monotherapy, calcium channel blockers (CCB) were the most commonly prescribed (19.3%). In dual therapy, the combination of CCB + ARB was most frequent (15.2%). Among triple therapy, CCB + ARB + diuretic was the most common combination (6.6%).

**TABLE 9:** Antihypertensive medications used in monotherapy and combination therapy.

Therapy type	Specific combination	Number of patients	Percentage (%)
A. Monotherapy			
	Calcium channel blocker (CCB)	38	19.3
	Angiotensin receptor blocker (ARB)	20	10.2
	Beta blocker (BB)	16	8.1
	ACE inhibitor (ACEi)	3	1.5
B. Dual therapy			
	CCB + ARB	30	15.2
	CCB + BB	15	7.6
	ARB + Diuretic	15	7.6
	BB + Diuretic	10	5.1
	CCB + Diuretic	8	4.1
	ACEi + Diuretic	4	2.0
	ARB + BB	4	2.0
	ACEi + CCB	3	1.5
	BB + ACEi	1	0.5
C. Triple therapy			
	CCB + ARB + Diuretic	13	6.6
	CCB + BB + Diuretic	8	4.1
	ACEi + BB + Diuretic	7	3.6
	ARB + BB + Diuretic	3	1.5
D. Quadruple therapy			
	CCB + ARB + BB + Diuretic	2	1.0
	CCB + ACEi + BB + Diuretic	1	0.5

### 3.5 WHO Prescribing Indicators

The average number of drugs prescribed per encounter was 4.05 ± 1.60 (mean ± SD). Drugs prescribed by generic name accounted for 34.4% of the total prescriptions. Antibiotics were prescribed in 14.2% of encounters, while injections were used in 7.6% of encounters. A total of 79.9% of the prescribed drugs were from the Essential Medicines List (Table 10).

**TABLE 10:** Assessment of WHO core prescribing indicators.

WHO prescribing indicator	Current study	WHO reference standard
Average number of drugs per encounter	4.05 ± 1.60 drugs	< 2.0 drugs
Percentage of drugs prescribed by generic name	34.4%	100%
Percentage of encounters with an antibiotic	14.2%	20%–26.8%
Percentage of encounters with an injection	7.6%	13.4%–24.1%
Percentage of drugs from Essential Medicines List	79.9%	100%

*Note:* Percentage of drugs from EML was calculated based on Nepal's National List of Essential Medicines, 2021 [35].

### 3.6 Association Between Antihypertensive Therapy and Other Factors

A statistically significant association was observed between therapy type and both JNC‐7 classification and age group (*p* < 0.001). Patients with higher blood pressure stages, particularly Stage 2 hypertension, were more likely to receive dual or polytherapy compared to those with normal or prehypertensive status, who predominantly received monotherapy. Similarly, older age groups showed a greater tendency toward combination therapy, with polytherapy being more common among patients aged ≥ 70 years, while younger patients were mainly managed with monotherapy. In contrast, no significant association was found between gender and therapy pattern (*p* = 0.530), indicating that treatment choice was not influenced by sex (Table 11). Overall, the results suggest that antihypertensive prescribing patterns were primarily guided by disease severity and age rather than gender.

**TABLE 11:** Association between JNC‐7 stage and antihypertensive therapy pattern (*N* = 197).

Variable	Monotherapy (*n*)	Dual therapy (*n*)	Polytherapy (*n*)	Total	*p*
JNC‐7 classification					
Normal	1	0	0	1	**< 0.001** *
Prehypertension	18	0	0	18	
Stage 1 hypertension	47	34	7	88	
Stage 2 hypertension	11	52	27	90	
Age group (years)					
18–29	11	3	0	14	**< 0.001** *
30–39	27	3	2	32	
40–49	21	18	2	41	
50–59	11	31	4	46	
60–69	5	22	11	38	
≥ 70	2	9	15	26	
Gender					
Male	37 (36.6)	48 (47.5)	16 (15.8)	101 (100)	0.530
Female	40 (41.7)	38 (39.6)	18 (18.8)	96 (100)	

*p < 0.05.

## 4 Discussion

The mean age of patients in this study was 51.58 ± 13.19 years, with the highest proportion in the 50–59 years' age group (23.9%). This finding is consistent with other studies from Nepal and India. Karki et al. reported a mean age of 59.7 ± 12.3 years among hypertensive patients in Palpa, Nepal [36], while Yadav et al. found that 77% of their study population belonged to the 40–60 years age group in a rural Nepali setting [37]. The age distribution reflects a well‐established epidemiological pattern: hypertension prevalence increases with advancing age due to vascular changes, cumulative exposure to risk factors, and longer disease duration [38]. The lower representation of younger age groups (18–29 years: only 7.1%) is expected, as hypertension in younger populations is less common and often secondary to other conditions.

Regarding educational status, more than one‐third of participants (39.1%) had secondary level education, while 22.3% were illiterate. This educational profile is typical for a rural, resource‐limited setting like Jumla, where access to higher education remains limited. The high proportion of housewives (37.6%) and farmers (28.4%) reflects the agrarian economy of Karnali Province [39]. From a clinical perspective, lower educational levels may be associated with poor health literacy, reduced medication adherence, and delayed healthcare seeking. These sociodemographic characteristics suggest that hypertension management in this population is influenced not only by biological factors but also by social determinants such as education, occupation, and health literacy [40]. This underscores the need for tailored patient education and community‐based interventions in rural Nepal.

The mean systolic blood pressure (155.91 ± 12.98 mmHg) and diastolic blood pressure (95.27 ± 6.46 mmHg) indicate that a majority of patients had uncontrolled or poorly controlled hypertension. According to JNC‐7 classification, 45.2% were in Stage 1 and 44.7% in Stage 2 hypertension, while only 0.5% had normal blood pressure. The high proportion of Stage 2 hypertension (44.7%) is concerning, as it suggests either delayed diagnosis, inadequate treatment, poor medication adherence, or a combination of these factors. This finding is comparable to Benur et al., who reported that 45.5% of their patients presented with Stage 2 hypertension [41]. The high proportion of Stage 2 hypertension reflects a critical gap in early detection and optimal control, which may increase the risk of cardiovascular complications such as stroke, myocardial infarction, and chronic kidney disease. This highlights the need for aggressive treatment strategies and regular follow‐up.

Regarding BMI classification, a majority of patients were either overweight (41.1%) or obese (33.5% combined across all obesity classes). Only 22.8% had normal BMI, and 2.5% were underweight. This high prevalence of overweight and obesity is a major modifiable risk factor for hypertension and cardiovascular disease. The finding aligns with the global trend of rising obesity in low‐ and middle‐income countries, including Nepal [42]. A positive family history of hypertension was reported by 64.0% of participants which is very similar to the study by Kunnas et al. [43] highlighting the strong genetic and familial predisposition to hypertension and underscoring the importance of screening first‐degree relatives [44].

Diabetes mellitus was the most common comorbidity (22.8%), followed by dyslipidemia (19.8%) and chronic kidney disease (11.7%). This pattern is consistent with Karki et al., who reported diabetes in 38.4% of their patients [36]. The presence of chronic kidney disease (11.7%) and ischemic heart disease (9.1%) indicates that a substantial number of patients already have target organ damage, necessitating aggressive blood pressure control. In this study, calcium channel blockers (CCBs) were the most frequently prescribed antihypertensive class (32.8%), followed by ARBs (24.6%), diuretics (19.3%), beta‐blockers (17.6%), and ACE inhibitors (5.6%). This finding is consistent with several studies from South Asia. Karki et al. in Nepal reported CCBs as the most prescribed class (66.5%) [36], while Singh et al. in Northern India found CCBs (25.1%) and ARBs (21.2%) to be most common [45]. Similarly, Sharma et al. and Vummareddy et al. both reported CCBs as the leading class in their Indian studies [46]. The predominance of CCBs, particularly amlodipine, can be explained by their efficacy in reducing blood pressure across all severity levels, favorable side effect profile, lack of adverse metabolic effects, wide availability, affordability, and recognition as a valid first‐line option in JNC 7 and JNC 8 guidelines. However, the relatively lower use of diuretics (19.3%) warrants consideration, particularly because dietary sodium exposure can influence blood pressure and response to antihypertensive therapy. Reduced sodium intake has been consistently associated with reductions in blood pressure in randomized trials and meta‐analyses [47, 48]. The present study did not collect sufficiently detailed individual‐level information on dietary sodium intake or the clinical rationale underlying drug selection; therefore, the lower use of diuretics cannot be interpreted as inappropriate prescribing on the basis of prescription frequency alone. Similarly, ACE inhibitors accounted for only 5.6% of prescriptions compared with 24.6% for ARBs. ACE inhibitors have demonstrated reductions in cardiovascular morbidity and mortality in several large randomized‐trial meta‐analyses, although comparative evidence between ACE inhibitors and ARBs is not entirely consistent across analyses [49]. The reasons for the relatively low utilization of ACE inhibitors in our setting cannot be determined from the present dataset because information on contraindications, previous treatment, adverse effects, renal function, electrolyte status, and prescriber preference was not systematically available.

Among individual drugs, amlodipine was the most prescribed (30.3%), followed by telmisartan (17.1%) and atenolol (13.4%). This finding mirrors Radhika et al., where amlodipine accounted for 53.2% of prescriptions [1], and Karki et al., where CCBs (primarily amlodipine) were prescribed in 66.5% of patients [36]. The WHO DDD analysis revealed that amlodipine contributed the highest total DDDs (3390), followed by telmisartan (1890) and atenolol (980). Interestingly, ramipril showed a relatively high DDD (1140) despite a lower prescription frequency (19 prescriptions), indicating that it was prescribed for longer durations or at higher strengths. Notably, telmisartan was prescribed at an average daily dose of 40 mg, exactly matching the WHO DDD, unlike Singh et al. who reported telmisartan prescribed at 46.15 mg (above WHO DDD) [45]. The dominance of amlodipine reflects a shift toward newer, well‐tolerated agents; however, this trend should be balanced with cost considerations and guideline adherence to ensure rational prescribing.

Regarding therapy patterns, dual therapy was the most common treatment pattern (43.7%), followed by monotherapy (39.1%), triple therapy (15.7%), and quadruple therapy (1.5%). The mean number of antihypertensive drugs per patient was 1.82 ± 0.87. This finding differs from some studies; Karki et al. reported combination therapy in 54.5% with a mean of 1.7 drugs per patient [36], while Singh et al. and Yadav et al. reported monotherapy as predominant (59.1% and 69%, respectively) [45]. The preference for dual therapy in this study is clinically appropriate because combination therapy provides a synergistic effect, allows the use of lower doses with fewer side effects, simplifies regimens, and is recommended by most guidelines for patients with Stage 2 hypertension or those not achieving target on monotherapy.

There was a highly statistically significant association between JNC‐7 stage of hypertension and antihypertensive therapy pattern (*p* < 0.001). Patients with Stage 2 hypertension were more likely to receive dual therapy and polytherapy compared to patients with lower stages. This finding is clinically expected, as patients with more severe hypertension require more intensive antihypertensive regimens to achieve blood pressure control [50]. A highly statistically significant association was observed between age group and antihypertensive therapy pattern (*p* < 0.001). Older patients (≥ 60 years) were more likely to receive dual therapy and polytherapy compared to younger patients. This is consistent with the higher prevalence of long‐standing hypertension, increased comorbidities, and greater cardiovascular risk in the elderly population. No statistically significant association was found between gender and antihypertensive therapy pattern (*χ* ^2^ = 1.271, df = 2, *p* = 0.530). Male and female patients were equally likely to receive monotherapy, dual therapy, or polytherapy. This finding suggests that treatment decisions at KAHS are not influenced by patient gender, reflecting equitable prescribing practices.

The average number of drugs per prescription (including all therapeutic categories) was 4.05, which is substantially higher than the WHO reference standard of less than 2.0 drugs per encounter. Comparing with other studies, Nachiya et al. reported 7.56 drugs per prescription [51], Radhika et al. reported 5.64 [1], and Singh et al. reported 4.0 [45]. The high average number of drugs per prescription in this study indicates significant polypharmacy, which can be attributed to the high prevalence of comorbidities requiring multiple medications, use of multiple antihypertensive agents (mean 1.82), prescribing of gastro protective agents (e.g., pantoprazole) with multiple other drugs, and adjunctive therapies including vitamins, supplements, and analgesics. This level of polypharmacy (mean 4.05 drugs per prescription) is approximately double the WHO recommended standard, indicating a substantial deviation that may increase the risk of drug interactions and reduce adherence. Although polypharmacy may be clinically justified in patients with multimorbidity, a higher medication burden increases the complexity of treatment and highlights the importance of regular medication review. In patients with persistent suboptimal BP despite multiple antihypertensive agents, assessment of adherence, treatment response, dosing, lifestyle factors, and potential opportunities for regimen simplification may help distinguish appropriate therapeutic intensification from potentially avoidable medication burden.

Only 34.4% of drugs were prescribed by generic name, which is far below the WHO reference standard of 100% [52]. Comparing with other studies, Radhika et al. reported an exceptionally high generic prescribing rate of 89.2%, which may reflect a strong institutional policy promoting generic prescribing at their site [1]. With only 34.4% generic prescribing, nearly two‐thirds of prescriptions rely on brand names, suggesting a significant and measurable economic burden on patients in a low‐income setting. The low generic prescribing rate in this study can be explained by prescriber habits and preferences for branded products, pharmaceutical marketing influence promoting brand names, limited awareness of cost differences between generic and brand‐name drugs, lack of generic prescribing policies at KAHS, and patient perception that branded drugs are superior. The consequences of low generic prescribing include increased out‐of‐pocket expenses for patients, higher healthcare costs, reduced medication adherence due to affordability issues, and inefficient use of limited healthcare resources. The extremely low rate of generic prescribing represents a major deviation from rational prescribing principles and is likely the single most important modifiable factor identified in this study.

A total of 79.9% of prescribed drugs were from Nepal's National Essential Medicines List (EML), which is below the WHO standard of 100%. Comparing with other studies, Karki et al. reported excellent EML adherence of 98.7% [36], and Singh et al. reported 74% [45]. While 79.9% is a moderate adherence rate, there is room for improvement. The 20.1% of drugs not on the EML likely include newer, more expensive agents, fixed‐dose combinations, or drugs not yet added to the national list. Suboptimal adherence to the Essential Medicines List suggests gaps in institutional prescribing policy and formulary enforcement, emphasizing the need for stronger governance and accountability mechanisms. Regular updates of the hospital formulary and prescriber education on EML content could improve adherence. Antibiotics were prescribed in 14.2% of encounters, which falls within or slightly below the WHO reference range of 20%–26.8% (lower is generally better to prevent antimicrobial resistance). Nachiya et al. reported antibiotic use in 14.9% of encounters, which is very similar to this study [51]. This reasonable antibiotic prescribing rate suggests that unnecessary antibiotic use is not a major problem at KAHS; however, ongoing antimicrobial stewardship is still recommended.

## 5 Conclusion

This study demonstrates that antihypertensive prescribing at KAHS, Jumla, is clinically appropriate in drug selection (CCBs and ARBs) and injection use. However, it significantly deviates from WHO rational drug use standards, with critical deficits in generic prescribing (34.4% vs. 100%) and excessive polypharmacy (4.05 vs. < 2.0 drugs per prescription). These gaps directly increase patient costs and health risks. Addressing them through targeted interventions could substantially improve the quality, affordability, and safety of hypertension care in this remote rural setting.

## Author Contributions

**Bijay Aryal:** supervision, writing – review and editing, conceptualization. **Birendra Kumar Yadav:** conceptualization, methodology, data curation, formal analysis, writing – review and editing, writing – original draft. **Biswash Sapkota:** methodology, writing – original draft, formal analysis. **Surakshya Adhikari:** writing – review and editing.

## Funding

The authors have nothing to report.

## Ethics Statement

Ethical approval for this study was obtained from the Institutional Review Committee (IRC) of Karnali Academy of Health Sciences (KAHS), Jumla (Ref. No.: 2025/068) prior to the commencement of the study.

## Conflicts of Interest

The authors declare no conflicts of interest.

## Data Availability

The data that support the findings of this study are available on request from the corresponding author.
